# Genetic parameters of white striping in relation to body weight, carcass composition, and meat quality traits in two broiler lines divergently selected for the ultimate pH of the pectoralis major muscle

**DOI:** 10.1186/s12863-016-0369-2

**Published:** 2016-04-19

**Authors:** Nabeel Alnahhas, Cécile Berri, Marie Chabault, Pascal Chartrin, Maryse Boulay, Marie Christine Bourin, Elisabeth Le Bihan-Duval

**Affiliations:** URA,INRA, 37380 Nouzilly, France; Syndicat des Sélectionneurs Avicoles et Aquacoles Français (SYSAAF), Centre INRA Val de Loire, Unité de Recherches Avicoles, F-37380 Nouzilly, France; Institut Technique de l’Aviculture (ITAVI), Centre INRA Val de Loire, F-37380 Nouzilly, France

**Keywords:** White striping, Genetic parameters, Muscle growth, Ultimate pH, Broilers

## Abstract

**Background:**

White striping (WS) is an emerging quality defect with adverse consequences for the sensorial, technological, and nutritional qualities of breast meat in broiler chickens. The genetic determinism of this defect is little understood and thus the aim of the study presented here was to estimate the genetic parameters of WS in relation to other traits of economic importance such as body weight, carcass composition, and technological meat quality in an experimental population consisting of two divergent lines selected for high (pHu + line) or low (pHu- line) ultimate pH (pHu) of the pectoralis major (p. major) muscle.

**Results:**

The incidence of WS in the whole population was 50.7 %, with 36.7 % of broilers being moderately and 14 % being severely affected. A higher incidence of moderate (*p* < 0.001) and severe (*p* < 0.0001) WS was observed in the pHu + line, and strong genetic determinism (h^2^ = 0.65 ± 0.08) was evidenced for WS in the studied lines. In addition, WS was significantly genetically correlated with body weight (r_g_ = 0.33 ± 0.15), and breast meat yield (0.68 ± 0.06), but not with the percentage of leg or abdominal fat. Increased body weight and breast muscle yield were significantly associated with increased incidence and severity of WS regardless of the line. Significant r_g_ were observed between WS and several meat quality traits, including breast (0.21 ± 0.08) and thigh (0.31 ± 0.10) pHu, and breast cooking loss (0.30 ± 0.15). WS was also strongly genetically correlated with the intramuscular fat content of the pectoralis major muscle (0.64 ± 0.09), but not with the lipid oxidation index of this muscle.

**Conclusions:**

This study highlighted the role of genetics as a major determinant of WS. The estimated genetic correlations showed that WS was more highly related to muscle development than to the overall growth of the body. The positive genetic association reported in this study between WS and muscle pHu indicated a possible relationship between the ability of muscle to store energy as a carbohydrate and its likelihood of developing WS. Finally, the strong genetic determinism of WS suggested that selection can be an efficient means of reducing the incidence of WS and of limiting its undesirable consequences on meat quality in broiler chickens.

## Background

The worldwide demand for poultry meat is constantly increasing, mainly because of its low price, ease and diversity of preparation, dietary and nutritional properties, and the fact that poultry meat production and consumption are not faced with obstacles of traditional or religious nature [1]. According to projections of the FAO, global poultry meat production and consumption are expected to increase by 1.8 % per annum between 2007 and 2050, which is considerably more than the expected increase in pork production and consumption (0.8 % per year) [2]. This increased demand could only be met by increased levels of production, putting more pressure on the poultry industry to produce birds with higher growth rates and feed efficiency. Intensive genetic selection has been the method of choice for the industry to improve these traits. Havenstein [3] estimated that 85 to 90 % of the change in growth rate observed over the last 50 years was due to genetic selection, while the remaining 10 to 15 % of the observed improvement in this trait was due to improved nutritional strategies. However, producing broilers capable of reaching market weight three times faster and with a third of the amount of feed [3] is not without consequences for animal physiology and meat properties. White striping (WS), an emerging non-infectious quality defect characterized by white striations appearing on broiler fillets and thighs parallel to the direction of muscle fibers, has recently been associated with high growth rate [4] and breast meat yield [5] in broilers. Other production factors such as sex and feeding regimen do not seem to play a major role in the incidence of this phenomenon [6]. Recent studies reported that the incidence of WS is far from negligible, with an estimate of 12 % in commercial conditions [5] and over 50 % in experimental conditions [4]. This defect leads to the rejection of the most expensive part of the carcass (i.e. the fillet) and affects purchasing decisions with adverse economic consequences [7]. Beyond the deleterious impact on the visual appearance and nutritional value of products [7, 8], WS affects several breast meat quality parameters including color and water holding capacity [5, 6]. For all these reasons, there is an increasing need to develop strategies to eradicate or at least reduce the incidence and severity of WS in modern commercial broilers. In this study we took advantage of the availability of two broiler lines divergently selected for breast meat ultimate pH [9] and affected by WS. As they are issued from a commercial fast-growing line, they are relevant to study the genetic determinism of WS and its relationships with growth, muscle development, and a wide range of meat quality traits. In addition, this unique model allows to investigate the potential implication reported in the literature [5, 10] of muscle ultimate pH on the incidence of WS.

## Results and discussion

### Phenotypic characterization

#### Incidence and severity of white striping

**Fig. 1:**
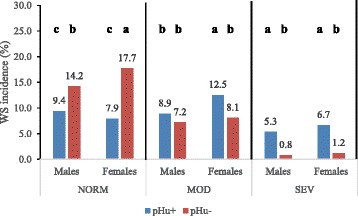
Incidence of white striping (WS) per line and sex. pHu + = broiler line selected for high value of ultimate pH; pHu- = broiler line selected for low value of ultimate pH. NORM = normal breast fillets; MOD = breast fillets moderately affected by white striping; SEV = breast fillets severely affected by white striping. Observed frequencies per line and sex have been compared within each category of WS. ^a-c^different letters indicate significant difference (p < 0.05) within each WS category

The incidence of white striping was determined by line and sex (Fig. 1). When totaling lines and sexes (*n* = 1349), 36.7 % of the fillets were categorized as moderately affected (MOD) and 14 % as severely affected (SEV), making a total of 50.7 %, which is in line with previously reported findings obtained in experimental conditions [4]. Frequencies of normal breast fillets were higher in the pHu- than in the pHu + line regardless of the sex, the difference being more pronounced in females than in males. Within the pHu- line, the proportion of normal fillets was higher in females than in males. Females of the pHu + line presented higher frequency of moderately white striped breast fillets compared to females of the pHu- line, while males of the two lines showed similar incidences. Finally, the incidence of severe white striping was higher in the pHu + than in the pHu- line for both sexes. The higher incidence of moderate (*p* < 0.001) and severe (*p* < 0.0001) WS observed in the pHu + line is in line with previous results that showed that white striped breast fillets were characterized by higher pHu than normal breast fillets [5, 10]. The positive association reported between breast muscle pHu and the increased degree of WS may be due to the fact that birds with the highest degree of WS also exhibited the highest breast muscle yield (BMY) [10]. This latter trait has already been shown to be negatively related to muscle glycogen reserve and positively related to pHu in broilers [11]. In a previous work [9], we have shown that despite a similar growth rate, the pHu + line exhibited higher BMY compared to the pHu- line, which could partly account for the higher incidence of WS in the pHu + line.

#### Body weight and carcass composition in relation to white striping

Regardless of the line, the MOD and SEV categories of WS were characterized by increased body weight (BW) and BMY compared to the NORM category (Table 1). These findings are in accordance with those of Kuttappan et al. [4] who found a higher degree of WS in birds fed a high energy diet, resulting in higher BW and BMY. According to these authors, enhanced growth rate and inadequate capillary development in breast muscles due to intensive selection could have resulted in a damaged muscular structure which manifests visually as WS. Our results also confirmed findings of Bauermeister et al. [8] and Petracci et al. [5] who reported that high yielding broilers were characterized by higher incidence and greater severity of WS than moderate yield type broilers. Our results therefore confirmed the unfavorable association between growth and breast muscle development, and the incidence of WS. Our results concerning the development of breast muscles indicated that the presence of WS was specifically associated with increased yield of the pectoralis major muscle (*p* < 0.0001), but not with that of the pectoralis minor muscle (*p* = 0.92). Our results also highlighted that WS is not related to abdominal fat percentage (AFP) or to leg percentage (LEGP) in these two lines.

**Table 1:** Intra-line effects of white striping on body traits expressed as LSMeans ± Standard error

		pHu+			pHu-		
	N	NORM (Nmax = 234)	MOD (Nmax = 289)	SEV (Nmax = 162)	NORM (Nmax = 431)	MOD (Nmax = 206)	SEV (Nmax = 27)
BW (g)	1,347	2,667 ± 16.0^b^	2,780 ± 14.5^a^	2,848 ± 19.2^a^	2,702 ± 11.9^b^	2,836 ± 17.0^a^	2,971 ± 47.0^a^
BMY (%)	1,340	20.3 ± 0.08^c^	21.2 ± 0.07^b^	21.9 ± 0.10^a^	20.0 ± 0.06^b^	20.8 ± 0.08^a^	20.9 ± 0.24^a^
PMY (%)	1,342	16.4 ± 0.07^c^	17.3 ± 0.06^b^	17.9 ± 0.09^a^	16.2 ± 0.05^b^	17.0 ± 0.07^a^	17.1 ± 0.21^a^
PmY (%)	1,339	3.9 ± 0.02	3.9 ± 0.01	3.9 ± 0.02	3.7 ± 0.01	3.8 ± 0.02	3.8 ± 0.05
AFP (%)	1,342	1.8 ± 0.02	1.8 ± 0.02	1.8 ± 0.02	1.9 ± 0.01	1.8 ± 0.02	1.7 ± 0.06
LEGP (%)	1,339	22.9 ± 0.07	22.7 ± 0.06	22.6 ± 0.08	22.4 ± 0.05	22.3 ± 0.08	22.7 ± 0.21

*pHu +* broiler line selected for high value of ultimate pH, *pHu-* = broiler line selected for low value of ultimate pH

*NORM* normal breast fillets, *MOD* breast fillets moderately affected by white striping, *SEV* breast fillets severely affected by white striping. *BW* Body weight at 6 weeks, *BMY* Breast meat yield, *PMY* Pectoralis major yield, *PmY* Pectoralis minor yield, *AFP* Abdominal fat percentage, *LEGP* Leg percentage

Nmax indicates the maximum number of observations per category

^a,b,c^ Different superscripts in different columns indicate significant intra-line differences between categories of white striping

#### Meat quality in relation to white striping

The WS conditions affected several breast meat quality traits, but not necessarily in the same way in both lines (Table 2). Lightness (L*) was generally higher in white striped than in normal breast fillets. Significant differences were observed between the NORM and SEV categories in the pHu + line (*p* = 0.03), and between the NORM and MOD categories in the pHu- line (*p* = 0.005). Previous studies [5, 6] did not identify breast meat L* as a discriminating factor to evaluate the presence of WS. However, they reported increased yellowness (b*) and redness (a*) in white striped fillets compared to normal fillets, which was not the case in the experimental lines of our study. Breast meat drip loss (DL) and cooking loss (CL) increased with the degree of WS in the pHu + line but not in the pHu- line. It is well established that high levels of L*, DL, and CL are usually associated with low pHu value in chicken breast meat [5, 9]. In the present study, breast meat pHu increased slightly with the degree of WS in the pHu- line, while no effect on this trait was found in the pHu + line. Despite these findings, changes in L* (in both lines), DL, and CL (only in the pHu + line) were observed. This suggests that changes in meat quality parameters are likely to be associated with the presence of WS rather than a consequence of a change in pHu. These results tend to corroborate a previous study [5] that reported concomitant increases in pHu and CL in both raw and marinated severely white-striped fillets and concluded that the effect of WS on meat quality parameters was independent of that of the pHu. As showed by Petracci et al. [12], muscle degeneration resulting from WS decreases muscle content of contractile (i.e., functional) proteins, including myosin and actin, leading to reduced ability of muscles to bind and retain water. Within each line, our results indicated that variation in the incidence and severity of WS did not impact, pH at 15 min post-mortem (pH15), a* and b* color parameters nor curing-cooking yield (CCY), Warner-Bratzler shear force (SF), and thiobarbituric acid-reactive substance (TBARS) of breast meat.

**Table 2:** Intra-line effects of white striping on breast pectoralis major muscle traits expressed as LSMeans ± Standard error

		pHu+			pHu-		
	N	NORM (Nmax = 234)	MOD (Nmax = 289)	SEV (Nmax = 162)	NORM (Nmax = 431)	MOD (Nmax = 206)	SEV (Nmax = 27)
L*	920	45.2 ± 0.3^b^	45.9 ± 0.2^ab^	46.4 ± 0.3^a^	52.3 ± 0.2^b^	53.5 ± 0.2^a^	52.4 ± 0.7^ab^
a*	903	−0.21 ± 0.04	−0.18 ± 0.04	−0.24 ± 0.05	0.12 ± 0.03	−0.03 ± 0.04	−0.12 ± 0.13
b*	921	10.2 ± 0.09	10.4 ± 0.08	10.5 ± 0.11	12.1 ± 0.07	12.3 ± 0.09	12.1 ± 0.28
pH15	595	6.73 ± 0.008	6.73 ± 0.008	6.75 ± 0.009	6.67 ± 0.006	6.68 ± 0.008	6.67 ± 0.025
pHu	1,344	6.11 ± 0.008	6.13 ± 0.007	6.13 ± 0.01	5.66 ± 0.006^b^	5.69 ± 0.009^a^	5.72 ± 0.02^ab^
DL (%)	1,273	1.8 ± 0.08^b^	2.2 ± 0.08^ab^	2.3 ± 0.10^a^	4.0 ± 0.06	4.1 ± 0.09	4.1 ± 0.25
CL (%)	593	8.6 ± 0.20^b^	9.2 ± 0.18^ab^	9.5 ± 0.22^a^	10.6 ± 0.15	11.0 ± 0.18	11.8 ± 0.57
CCY (%)	556	86.0 ± 0.40	86.3 ± 0.36	86.2 ± 0.43	84.1 ± 0.31	82.9 ± 0.37	82.2 ± 1.16
SF (N/cm^2^)	594	11.3 ± 0.25	11.2 ± 0.23	11.0 ± 0.27	16.2 ± 0.19	15.8 ± 0.23	15.9 ± 0.72
IMF (%)	597	1.3 ± 0.03^c^	1.4 ± 0.03^b^	1.7 ± 0.04^a^	1.3 ± 0.03^b^	1.6 ± 0.03^a^	1.8 ± 0.10^a^
TBARS	592	0.27 ± 0.04	0.31 ± 0.03	0.35 ± 0.04	0.53 ± 0.03	0.47 ± 0.03	0.42 ± 0.10

*pHu +* broiler line selected for high value of ultimate pH, *pHu-* broiler line selected for low value of ultimate pH

*NORM* normal breast fillets, *MOD* breast fillets moderately affected by white striping, *SEV* breast fillets severely affected by white striping. L* Lightness a* = Redness, b* Yellowness, *pH15* pH at 15 min post-mortem, *pHu* Ultimate pH, *DL* Drip loss, *CL* Cooking loss, *CCY* Curing-cooking yield, *SF* Shear force, *IMF* Intramuscular fat content, *TBARS* Thiobarbituric acid reactive substances index expressed as mg of malonedialdehyde per kg of meat

Nmax indicates the maximum number of observations per category

^a,b,c^ Different superscripts in different columns indicate significant intra-line differences between categories of white striping

As already reported [4, 12, 13], the amount of intramuscular fat (IMF) increased with the degree of WS in both lines. It has been suggested that when the damage to the muscle tissue is acute or continuous, attempts to repair or regenerate the damaged zones may fail, leading to differentiation of the pluripotent stem cells of the muscle tissue into fibroblasts or adipocytes, which in turn lead to fibrosis and lipidosis. The occurrence of these phenomena accounts for the higher collagen and intramuscular fat content associated with WS, respectively [4, 12, 13]. Although the IMF content increased with the presence of WS in the present study, no effect of the WS condition was observed on breast meat lipid peroxidation, as measured by the TBARS index after 9 days of storage at 4 °C. The absence of effect on the TBARS index suggests that, despite the increase in IMF content of breast fillets, WS does not influence the storage ability of fresh meat.

### Estimation of genetic parameters

The descriptive statistics of traits (other than WS) included in the genetic analyses are summarized in Table 3. Estimates of heritability (h^2^) and genetic correlations (r_g_) between WS and body or meat quality traits in the whole population are presented in Table 4. Because the breast meat characteristics and the level of incidence of WS differed between the two lines, we also estimated the genetic correlations within each of the two lines (Table 4).

**Table 3:** Descriptive statistics of traits included in the genetic analyses

	N	Mean	SD	CV%	Min	Max
BW (g)	5,668	2,741	389	14.2	1,527	4,408
BMY (%)	5,436	20.2	1.4	7.1	15.0	25.8
PMY (%)	5,305	15.5	2.8	18.0	6.2	22.4
PmY (%)	5,271	3.9	0.8	21.7	1.3	7.8
AFP (%)	5,435	1.9	0.4	22.0	0.7	4.0
LEGP (%)	1,500	22.6	1.2	5.3	18.6	27.0
L*	4,120	48.9	4.1	8.4	35.1	59.8
a*	3,977	−0.07	0.7	-	−1.85	2.93
b*	4,123	10.7	1.6	14.8	5.8	15.9
pH15	597	6.71	0.09	1.3	6.42	7.02
pHu (breast)	5,459	5.90	0.22	3.7	5.29	6.65
pHu (thigh)	1,492	6.45	0.23	3.6	5.84	7.00
Log(DL)	1,293	0.7	0.5	74.6	−1.3	2.1
CL (%)	593	9.9	2.2	22.1	4.0	20.4
CCY (%)	557	84.9	4.1	4.5	70.1	94.3
SF (N/cm^2^)	594	13.6	3.5	25.6	5.7	24.1
IMF (%)	600	1.4	0.4	29.2	0.5	3.2
TBARS	593	0.40	0.39	97.5	0.02	3.76

*BW* Body weight at 6 weeks, *BMY* Breast meat yield, *PMY* Pectoralis major yield, *PmY* Pectoralis minor yield, *AFP* Abdominal fat percentage, *LEGP* Leg percentage. L* Lightness, a* Redness, b* Yellowness, *pH15* pH at 15 min post-mortem, *pHu* Ultimate pH, *DL* Drip loss, *CL* Cooking loss, *CCY* Curing-cooking yield, *SF* Shear force, *IMF* Intramuscular fat content, *TBARS* Thiobarbituric acid reactive substances index expressed as mg of malonedialdehyde per kg of meat

**Table 4:** Genetic parameters of white striping in relation to body weight, carcass composition and breast meat quality

		Total population		Intra-line	
	*σ*_*p*_^2^	h^2^ ± se	r_g_ ± se	r_g_ ± se (pHu+)	r_g_ ± se (pHu-)
BW	69125	**0.20 ± 0.04**	**0.33 ± 0.15**	**0.26 ± 0.06**	0.14 ± 0.29
BMY	1.89	**0.60 ± 0.04**	**0.68 ± 0.06**	**0.74 ± 0.08**	**0.62 ± 0.15**
PMY	1.28	**0.57 ± 0.04**	**0.73 ± 0.07**	**0.76 ± 0.05**	**0.81 ± 0.08**
PmY	0.14	**0.38 ± 0.04**	**0.48 ± 0.16**	0.06 ± 0.13	0.02 ± 0.21
AFP	0.15	**0.72 ± 0.10**	−0.12 ± 0.10	−0.09 ± 0.14	−0.17 ± 0 .17
LEGP	1.37	**0.61 ± 0.07**	0.02 ± 0.12	−0.10 ± 0.15	0.19 ± 0.19
L*	10.98	**0.59 ± 0.05**	0.17 ± 0.10	**0.45 ± 0.13**	**0.62 ± 0.10**
a*	0.35	**0.37 ± 0.04**	−0.07 ± 0.13	0.19 ± 0.20	0.19 ± 0.45
b*	1.53	**0.49 ± 0.04**	0.10 ± 0.11	**0.40 ± 0.12**	−0.01 ± 0.25
pH15	0.01	**0.37 ± 0.09**	0.21 ± 0.16	0.23 ± 0.12	0.27 ± 0.28
pHu (breast)	0.02	**0.55 ± 0.04**	**0.21 ± 0.08**	0.18 ± 0.13	0.19 ± 0.22
pHu (thigh)	0.03	**0.63 ± 0.08**	**0.31 ± 0.10**	**0.31 ± 0.14**	0.28 ± 0.22
Log(DL)	0.12	**0.57 ± 0.10**	0.00 ± 0.12	**0.34 ± 0.13**	0.23 ± 0.27
CL	4.45	**0.45 ± 0.09**	**0.30 ± 0.15**	**0.56 ± 0.26**	**0.71 ± 0.20**
CCY	15.86	**0.18 ± 0.06**	−0.06 ± 0.22	−0.37 ± 0.27	**−0.77 ± 0.23**
SF	7.59	**0.53 ± 0.08**	−0.21 ± 0.14	0.06 ± 0.30	−0.19 ± 0.35
IMF	0.18	**0.83 ± 0.09**	**0.64 ± 0.09**	**0.63 ± 0.11**	**0.51 ± 0.25**
TBARS	0.15	**0.20 ± 0.06**	0.00 ± 0.21	0.31 ± 0.30	−0.38 ± 0.28
WS	0.54	**0.65 ± 0.08**	-	-	-

*σ*_*p*_^2^ = Phenotypic variance estimated by the model as the sum of the genetic variance, the maternal environmental variance (for BW), and the residual variance. h^2^ ± se = Estimated heritability ± standard error of the estimate; r_g_ ± se = Estimated genetic correlation with WS ± standard error of the estimate. *BW* Body weight at 6 weeks, *BMY* Breast meat yield, *PMY* Pectoralis major yield, *PmY* Pectoralis minor yield, *AFP* Abdominal fat percentage, *LEGP* Leg percentage, L* Lightness, a* Redness, b* Yellowness, *pH15* pH at 15 min post-mortem, *pHu* Ultimate pH, *DL* Drip loss, *CL* Cooking loss, *CCY* Curing-cooking yield, *SF* Shear force, *IMF* Intramuscular fat content, *TBARS* Thiobarbituric acid reactive substances index, *WS* White striping.

Estimates in bold are significantly different from 0 based on their confidence intervals

The estimated heritability of WS (0.65) indicates that genetics is a major determinant of this defect in the studied lines. This estimate is considerably greater than the only estimates reported by Bailey et al. [14] in two commercial pure lines of broiler chickens selected for high (h^2^ = 0.34) or moderate (h^2^ = 0.18) breast meat yield. This difference in magnitude may be related to differences in genetic background (i.e., different base populations), selection criteria (breast meat pHu vs. breast muscle yield) and methods of estimation. While Bailey et al. [14] estimated WS heritability on a 4-point observed scale, in the present study h^2^ was estimated on an underlying continuous scale, which can result in higher values. Using the approximation developed by Dempster and Lerner [15] in the case of a binary trait, a heritability of 0.65 on this underlying scale would correspond to an estimate of 0.41 on the observed scale for an incidence of 0.50 (which is the case for WS if we group MOD and SEV categories together). Estimates of h^2^ calculated in the present study for body weight, carcass composition (i.e., breast meat yield, leg yield, and abdominal fat percentage), and meat quality parameters were in the range reported in our previous paper [9].

In accordance with the phenotypic findings of the current study, WS was found to be positively genetically correlated with BW and BMY at slaughter. In the whole population, the genetic correlation (r_g_) with the latter trait was twice as high as with the former (r_g_ = 0.68 vs. 0.33). In addition, WS was also more strongly correlated with the percentage of pectoralis major muscle than with that of pectoralis minor muscle (r_g_ = 0.73 vs. 0.48). The strong positive genetic correlations between WS on one hand and BMY or pectoralis major yield (PMY) on the other hand were confirmed within each of the lines. By contrast, the genetic correlation between pectoralis minor yield (PmY) and WS was no longer significant when estimated separately in the pHu + and pHu- lines. For BW, the genetic correlation with WS was only significant in the pHu + line, due to the high standard error of the estimation in the pHu- line. The stronger genetic correlations found between the BMY and the percentage of pectoralis major muscle (r_g_ = 0.91 ± 0.01) than found between the BMY and the percentage of pectoralis minor muscle (0.58 ± 0.06) indicates that breast meat yield is mainly determined by the percentage of pectoralis major muscle, which is in agreement with the findings of Reddish and Lilburn [16]. According to these authors, the width and thickness of the pectoralis major muscle are particularly targeted by selection for high BMY, which is entirely consistent with the greater occurrence of WS observed in the pectoralis major compared to the pectoralis minor muscle [17]. The positive r_g_ reported in the present study for body weight and muscle development with WS indicate that the incidence and the degree of this defect can be expected to be higher when the selection focus is on higher BW and BMY. Our results also highlight that WS is more influenced by genetic progress in breast meat yield (especially that of pectoralis major muscle) than in growth rate. Interestingly, WS was not found to be significantly correlated with AFP nor with LEGP in the whole population and within each of the two divergent lines. However, and in accordance with the phenotypic findings of the current study, WS was highly correlated with the IMF content of the pectoralis major muscle in the whole population (0.64 ± 0.09) and within each of the two lines. Moreover, in the present study the IMF was found to be far more heritable (h^2^ = 0.83) than the IMF reported in the slow-growing chicken genotypes used in Label Rouge type production (h^2^ = 0.18) in France [18]. Such differences in heritability may be due to differences in IMF content and the variability observed between the slow- and the fast-growing line investigated in that study, the latter containing 40 % more lipids on average than the former, certainly as the result of the intensive selection on growth rate and muscle development, and the presence of WS. The high genetic correlation between WS and IMF also indicates that the latter measurement could be used as a valuable quantitative indirect criterion of selection against WS.

In the whole population, WS was not correlated with breast pH15, L*, a*, b*, DL, CCY, SF, and TBARS. A significant correlation was only found with breast CL (r_g_ = 0.30 ± 0.15) indicating increased water loss after cooking with increased degree of WS. Interestingly, intra-line analyses revealed strong positive correlations that were not observed or at lesser level when considering one unique population. It was the case for L* and CL that were both strongly positively related to WS in the pHu + and pHu- lines, b* and DL that were positively correlated to WS in the pHu + line, and CCY that was highly negatively correlated in the pHu- line. The positive genetic correlation reported between the WS condition and CL is consistent with previous results that showed increased juice loss during cooking [19]. These authors also reported decreased marinade uptake in white-striped fillets compared to normal fillets. As observed in the total population, no significant correlation was found between WS and a*, pH15, SF, and TBARS within each of the two divergent lines.

In the whole population, moderate but significant positive genetic correlations were estimated between WS and muscle pHu (r_g_ = 0.21 ± 0.08 and 0.31 ± 0.10 for breast and thigh muscle, respectively). Similar estimates were found within each of the two lines but, because of larger standard errors when separating the two lines, only the correlation between WS and thigh pHu in the pHu + line remained significant. These positive correlations confirmed a possible link between the incidence of this defect and the energy status of the muscle during life. Indeed, increased pHu in the breast muscle of chickens reflects decreased levels of glycogen reserve [20]. Muscles containing low glycogen content before slaughter may thus be more susceptible to WS. Improvement of breast muscle mass and yield in broilers is mainly achieved by increasing muscle fiber size, which results in decreased muscle glycogen content [11]. Such structural and metabolic changes may in part be responsible for the emergence of white striping whose incidence is particularly high in heavy broiler production [5, 12]. Indeed, the chicken pectoralis major muscle is almost entirely made of fast-twitch glycolytic fibers [1, 21]. It is characterized by reduced capillary density [22] and by the predominance of the anaerobic glycolytic pathway for energy regeneration where glucose, originating mainly from the glycogen reserve of the muscle, is the only substrate used to generate energy [23]. In addition, we have recently shown that selection for increased breast muscle pHu was associated with reduced muscle capillary density [24]. It could therefore be hypothesized that in modern heavy broilers reduced muscular vascularization and glycogen reserve may compromise energy supply to muscle fibers. Such a condition could induce protein catabolism as an alternative pathway to produce energy, resulting in impaired muscle fiber development and functioning and, as a consequence, progressive replacement of muscle tissue by adipose and connective tissues during growth, as observed in white striped breast fillets.

## Conclusions

The present study highlighted the strong genetic determinism of the white striping condition in the studied lines. Positive genetic correlations were evidenced with breast meat yield and to a lesser extent with body weight, two traits that are selected intensively in modern broiler lines. The increased degree of white striping was also phenotypically and genetically associated with increased levels of intramuscular fat and to a lesser extent with cooking loss, which emphasized the negative impact of this defect on the nutritional value and processing ability of chicken breast meat. The positive relationship observed for white striping with muscle ultimate pH and yield suggests that both fiber hypertrophy and lack of energy reserve in muscle could in part be responsible for the emergence of this defect in heavy broiler lines and this requires further investigation to confirm the relationship. Given the heritability of white striping, selection may be proposed as an effective tool to reduce the occurrence of this defect in broilers. However, in view of the positive genetic correlations with breast meat yield and body weight, this would involve a compromise between the occurrence of white striping and the genetic progress achieved for these traits.

## Methods

### Birds and housing

The study was conducted on birds originating from two lines divergently selected for breast meat pHu according to a breeding scheme described in Alnahhas et al. [9]. White striping was evaluated on a total of 1349 broilers produced by 114 sires and 300 dams, originating from the 5^th^ and 6^th^ generations of divergent selection on the pHu of the pectoralis major muscle. Of this total, 685 birds (319 males and 366 females) were from the line selected for high breast pHu value (i.e., the pHu + line) and 664 birds (300 males and 364 females) from the line selected for low breast pHu value (i.e., the pHu- line). After hatching, day-old chicks were identified by wing tags, sexed, and vaccinated against Infectious Bronchitis. Birds from the two divergent lines were reared as a single population (with males and females of both lines mixed together) in a standard closed poultry house of the INRA experimental unit (PEAT, F-37380 Nouzilly, France). Broilers were reared under standard rearing practices, as described in Alnahhas et al. [9], and had *ad libitum* access to feed, and water during the rearing period.

### Slaughter and processing

At the age of 6weeks and after 8 h of feed withdrawal, birds were weighed and transported to the experimental slaughter house of PEAT. Slaughtering and processing were performed as described in Alnahhas et al. [9]. The day after slaughter, the right breast pectoralis major muscle was scored for white striping. The categories of WS were defined according to a modified version of the scale of Kuttapan et al. [7] to account for the lower degree of severity in our experimental population. The fillets were scored as normal (NORM) in the absence of WS, moderate (MOD) corresponding to score 1 (striation thickness < 1 mm) or severely affected (SEV), corresponding to score 1.5-2 (striation thickness ≥ 1 mm) of Kuttapan et al. [7]. Body composition and meat quality traits were determined through the measurement of several parameters, as described in Alnahhas et al. [9]. Breast meat yield (BMY), leg percentage (LEGP), and percentage of abdominal fat (AFP) were determined in relation to body weight (BW). Breast meat quality was evaluated on pectoralis major muscle through the measurement of pH at 15 min post-mortem (pH15), pHu, color parameters L*, a*, b*, drip (DL), and cooking (CL) loss, Warner-Bratzler shear force (SF) of cooked meat, and curing-cooking yield (CCY). Ultimate pH was also measured in the Sartorius muscle of thigh. Briefly, the pHu was measured 24 h post-mortem using a portable pH meter (model 506, Crison Instruments SA, Alella, Barcelona, Spain) by direct insertion of its glass electrode into the muscles. L*, a*, and b* color parameters were measured at the same time on the internal face of the muscle using a miniscan spectrocolorimeter (Hunterlab, Reston, VA, USA). DL was determined after 5-day storage at 2 °C of the entire muscle hanged and zip-locked in a plastic bag. CL was measured after cooking a vacuum-packed meat sample of 180 g in a water-bath (85 °C for 13 min). The SF of the cooked meat was then measured using an Instron universal testing instrument (Instron 5543, Instron S.A., Guyancourt, France). For each sample, measurement was performed on 3 adjacent strips (3 × 1 × 1 cm) of meat and the average of the maximum force necessary to shear the meat was recorded. CCY was measured on 60 g of minced muscle mixed with 20 % nitric salt solution for 24 h at 4 °C. Intramuscular fat (IMF) content was determined from samples of pectoralis major muscle, taken at slaughter and kept at −20 °C until analysis. After thawing overnight at 4 °C, samples were ground and the intramuscular fat content was determined from about 50 g of ground meat by near-infrared spectroscopy using a Nirflex N-500 spectrometer (Buchi, Rungis, France) as described in Chartrin et al.[25]. The level of lipid peroxidation was also evaluated by measuring the thiobarbituric acid-reactive substance (TBARS) index [26] in pectoralis major muscle samples, aged for 9 days at 2 °C then stored at −80 °C and thawed overnight at 4 °C. The TBARS index was expressed as mg of malonedialdehyde per kg of meat after thawing. The measurements of pH15, CL, CCY, SF, IMF, and TBARS were limited to birds from the 6^th^ generation of selection.

### Phenotypic characterization

The incidence of WS was calculated and compared between lines and sexes separately for each category of WS using the Chi-squared test implemented in the PROC FREQ of SAS [27]. Data inspection, elimination of outliers, and tests of normality for body and meat quality traits were performed using PROC UNIVARIATE of SAS [27] prior to analysis of variance. The model included the fixed main effects of number of hatch (H_i_, *i* = 1 to 4), sex (S_j_, *j* = 1 for male, 2 for female), and genetic line (L_k_, *k* = pHu+, pHu-). As hatch and generation were confounded, different hatch numbers were considered for the different generations. A term for WS intra line (WS(L)_kl_, *l* = 1 to 3) was also included in the model to analyze the effects of WS within each line independently from the effect of the pHu. The final model equation was as follows:

$$ {y}_{ijklm}={H}_i+{S}_j+{L}_k+WS{(L)}_{kl}+{e}_{ijklm} $$

where y_ijklm_ is the record of the m^th^ bird for the trait analyzed, and e_ijklm_ is a random residual assumed to follow a normal distribution e_ijklm_ ~ N(0, σ^2^). All other terms are as indicated above. The analyses were implemented using the GLM procedure of SAS software [27]. Means of different effects were separated and compared for significant differences using Tukey’s method, as implemented in the same procedure via the LSMEANS option. The accepted type I error was set at 5 %.

### Estimation of genetic parameters

In addition to the data collected at the 5^th^ and the 6^th^ generations, the genetic analysis included measurement of body weight, breast meat yield, pHu, color parameters (L*, a*, and b*), and percentage of abdominal fat acquired from the four previous generations [9]. The pedigree file included a total of 10,621 birds produced by 382 sires and 1011 dams. Of this total, 1640 birds (15.5 %) belonged to the base population (G0), 4295 birds (40.4 %) belonged to the pHu + line, and 4686 birds (44.1 %) to the pHu- line. Data inspection, elimination of outliers, and tests of normality for quantitative traits were performed using PROC UNIVARIATE of SAS [27]. A logarithmic transformation was applied to normalize the distribution of the drip loss data before running the genetic analysis. Descriptive statistics of all available data, recorded between the 1^st^ and the 6^th^ generations, for all traits included in the estimation of genetic parameters were generated using PROC MEANS of the same software.

Given that WS is measured as a categorical trait and that threshold models are one of the most used methodologies to analyze this kind of data, a series of bi-variate (a single quantitative trait with WS at a time) generalized linear mixed animal models was fitted to the data using the Gibbs sampling method as implemented in the software TM [28] for Threshold Model. In this methodology, the phenotypic expression of categorical trait is attributed to an underlying continuous normally distributed unobservable trait referred to as the liability [29]. The number of thresholds is defined as (m – 1), where m is the number of observed categories for the observed trait. When the liability for a bird exceeds a particular threshold, the corresponding category (i.e., the phenotype) is expressed [30]. The model equation that we implemented to analyze the liability to white striping or the quantitative traits measured in our population was the following:

$$ {y}_{ijkl}=\mu +{H}_i+{S}_j+{c}_k+{a}_l+{e}_{ijkl} $$

where y_ijkl_ is the record of the l^th^ individual (*l* =1 to 10,621 birds), μ the general mean of the population, H_i_ the fixed effect of the i^th^ hatch (*i* = 1 to 21 hatches), S_j_ the fixed effect of the j^th^ sex (*j* = 1 for male, 2 for female), c_k_ the random effect of the common maternal environment (*k* = 1 to 1011 dams) which was included in the model only for BW, a_l_ the direct additive genetic effect of the bird (*l* =1 to 10,621 birds), and e_ijkl_ a random error.

In the bi-variate analysis of the liability underlying WS (noted as 1) and the quantitative trait (noted as 2), the vectors of the residual terms had the following (co)variance structure $$ Var\left(\begin{array}{c}\hfill {e}_1\hfill \\ {}\hfill {e}_2\hfill \end{array}\right)={\oplus}_{k=1}^n{\boldsymbol{R}}_k $$ where **R**_**k**_$$ =\left(\begin{array}{cc}\hfill {\sigma}_{e1}^2\hfill & \hfill {\sigma}_{e12}\hfill \\ {}\hfill {\sigma}_{e21}\hfill & \hfill {\sigma}_{e2}^2\hfill \end{array}\right) $$, n is the number of birds and ⊕ is the direct sum operator. For the vectors of the additive genetic effects, the following (co)variance structure was assumed: $$ Var\left(\begin{array}{c}\hfill {a}_1\hfill \\ {}\hfill {a}_2\hfill \end{array}\right)=\boldsymbol{A}\otimes \boldsymbol{G} $$, where ⊗ is the direct product operator (i.e., the Kronecker product), **A** is the relationship matrix between the birds, and **G** is the matrix of the genetic additive (co)variance components with the following structure: **G**$$ =\left(\begin{array}{cc}\hfill {\sigma}_{g1}^2\hfill & \hfill {\sigma}_{g12}\hfill \\ {}\hfill {\sigma}_{g21}\hfill & \hfill {\sigma}_{g2}^2\hfill \end{array}\right) $$.

The total number of iterations used in the Gibbs sampler was 100,000 iterations in a single Markov Chain Monte Carlo (MCMC) chain. The first 20,000 iterations were discarded (i.e., burn-in iterations) and every 20 iterations (thinning interval) a sample was saved from the remaining 80,000 iterations. The 4000 estimations resulting from the sampling process were used to estimate the genetic parameters and their standard errors. For each one of the 4000 samples, heritability of the liability to WS or the quantitative trait was calculated as the ratio of the additive genetic variance over the sum of the genetic and residual variances i.e., ${h}^2=\frac{\sigma_g^2}{\sigma_g^2+{\sigma}_e^2}$. Then, the mean and standard deviation for the 4000 estimates were taken as the heritability estimate of the trait and its standard error. Genetic correlations were calculated at each iteration as the ratio of the additive genetic covariance between traits over the product of their genetic standard deviations i.e., ${r}_g=\frac{\sigma_{g12}}{\sqrt{\sigma_{g1}^2\times {\sigma}_{g2}^2}}$, and similarly to the heritability, the mean and standard deviation of the calculated correlations over all iterations were taken as the estimate and its standard error. Convergence of threshold models was tested by plotting the traces and running means of the posterior distributions. The convergence was also tested by the Heidelberger and Welch test as implemented in the boa package version 1.1.7-2 [31] under the R Statistical Environment [32].

To perform the intra-line estimation of genetic parameters, the same methodology as for the whole population was applied to a dataset containing only the pHu + line, or only the pHu- line. The purpose of the intra-line analysis was to detect potential differences between the two divergent lines in term of genetic relationships between white striping and other traits of interest in the present study.

### Ethics

The study was conducted on two divergent lines owned by INRA. All animal care and experimental procedures needed for the selection and the phenotyping of the two divergent lines (referenced as programs N°00881.02 and N°00880.02) were approved by the Ethics Committee for Animal Experimentation of Val de Loire registered under N° 19 by the National Committee.

### Consent to publish

Not applicable.

### Availability of data and materials

All the data that support the conclusions of the study are included in the paper.

## Abbreviations

AFP: abdominal fat percentage
BMY: breast muscle yield
BW: body weight
CCY: curing-cooking yield
CL: cooking loss
DL: drip loss
IMF: intramuscular fat
LEGP: leg percentage
MOD: moderately affected
NORM: normal
pH15: pH at 15 min post-mortem
pHu: ultimate pH
PMY: pectoralis major yield
PmY: pectoralis minor yield
SEV: severely affected
SF: Warner-Bratzler shear force
TBARS: thiobarbituric acid-reactive substance
WS: white striping
